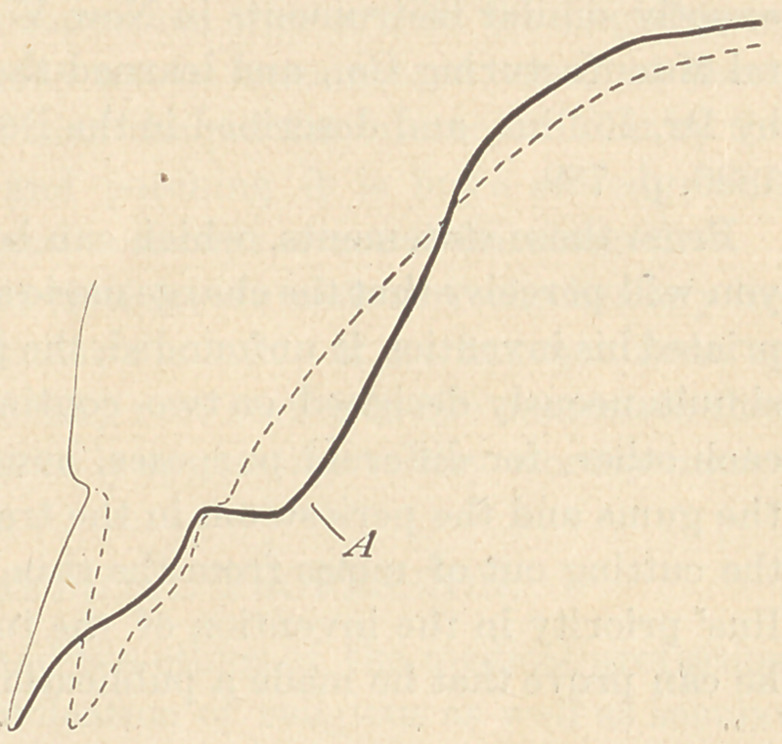# Current News and Opinion

**Published:** 1888-06

**Authors:** 


### Current News and Opinion.

### CONCERNING EROSION.

BY W. D. MILLER.

The question of the etiology of erosion (abrasion, denudation) is one which, however much it has been discussed, is little nearer a definite solution to-day than it was twenty years ago. Nor is it my object now to make any attempt at an explanation of the phenomenon. I simply desire to record an experiment which definitely settles the question as to whether or not erosion occurs in pulpless teeth. We have all seen pulpless teeth which presented extensive erosions, but we have not been able to say that these erosions were not produced while the pulp of the tooth was still alive, and, as far as I am aware, no one has succeeded in refuting beyond all doubt the assertion that erosion attacks only teeth with living pulps The following experiment is therefore of considerable value, inasmuch as it definitely settles the question at issue.

On the 7th of April, 1886, a piece of ivory was set, by means of cement, in the cavity of a right inferior bicuspid, where the loss of substance by erosion was so extensive that it would have exposed the pulp if the latter had not been protected by secondary dentine. The piece of ivory at the time it was set had the form represented by the dotted line in the figure. On the 23d of April, 1888, a little more than two years later, it presented the appearance seen at *a*, in the figure in cross section.

The piece was then removed for examination, and showed two very distinct parallel horizontal furrows. The surface had the very fine polish characteristic of abraded dentine. No one examining the piece of ivory would hesitate for a moment to pronounce it a typical case of erosion.

---

### THE CONSTRUCTION OF ARTIFICIAL DENTURES.

At the twentieth annual meeting of the Eighth District Dental Society, held in Buffalo, April 17 and 18, Dr. Geo. B. Snow made some valuable and practical remarks upon the proper method of proportioning artificial plates, those made of rubber, especially, that perfect and distinct enunciation may be secured. He said that if the cast of a jaw in which the natural teeth are in place be sawn longitudinally through the centre, and the contour of the palatine surface carefully studied, it will surprise most dentists. Nor will there be any great departure from the same general line, no matter what may be the shape of the mouth. A prominence will be seen just back of the anterior teeth, and it is against this that the tongue rests in the production of many of the sounds of articulate speech. When the teeth are extracted this prominence is soon lost, and it is not entirely due to the absence of the teeth that the imperfect articulation of edentulous persons is due. This prominence does not exist, and hence the tongue does not perfectly rest against the anterior portion of the jaw, as is necessary in giving the sounds of d, t, g, j, and others of the letters of the alphabet.

When an artificial plate is constructed, it is usual to make it as thin as possible at this very point, where it should be thick, and hence the speech of those using gold plates, especially, is usually very indistinct. The annexed imperfect cut will illustrate this. The heavy line is about that of the usual jaw with natural teeth, and at "A" is the prominence referred to. The dotted line is that of the ordinary artificial denture. The proper contour is obtained by adding to the thickness of the plate at the right point, until the natural contour is approached, and a prominence thus formed for the tougue to meet in articulate speech. If, then, the natural rugæ of the mouth can be reproduced, it will materially aid speech. But the plate should be made especially thick where it is usually thin, and the added material should be so shaped as to reproduce the natural prominent ridge. Those who have experimented in cases in which there was a very disagreeable sibilant sound, or hissing, in conversation, will remember that if they overcame the difficulty it was not by making the plate thin at the anterior portion of the vault, but by adding to its thickness. The objectionable sound is caused by the escape of air through the imperfect occlusion of the tongue with the anterior portion of the roof of the mouth. If additional thickness be given the denture at this point, it also adds to its strength just where rubber plates usually are weakest. Continuous gum plates usually give the clearest enunciation, because they are thick at the essential point, by the addition of the rugæ.

---

### CORRESPONDENCE.

*Editor Independent Practitioner :—*

The article by Dr. W. H. Rollins in the INDEPENDENT PRACTITIONER for April, 1888, page 212, induces me to make a short reply, which I beg of you to publish in your excellent Journal. The instruments which I exhibited in the Section of Dental and Oral Science of the Ninth International Medical Congress were not "circular," but "tubular" knives. I have used such instruments for the excision of moles from the skin of the face since 1884, and published a communication in the *Berliner Klinische Wochenschrift* for 1884, p. 386, on the extirpation of small round excrescences of the skin by means of quickly rotating hollow irons (*Schnell rotierende locheisen*). I take the liberty of sending you the number of the Journal referred to. A short time after I accepted the direction of the Dental Institute of the Berlin University, I had some tubular knives made for the dental engine according to my own design, at the dental depot of Paul Buss, of this city, and used them for the purpose stated above. In September,

1887, I exhibited the instruments at the International Congress, together with about thirty moles removed by their means. To my great astonishment I saw exactly similar instruments in New York, in the depot of the S S. White Dental Manufacturing Co., and learned there that they were designed in America by Dr. Rollins, and described in the December number of the *Dental Cosmos* for 1886, p. 789.

From these statements, which can be substantiated by the literary evidence, you will perceive that the charge made against me by Dr. Rollins of having appropriated his invention is unfounded, the fact seeming to be that the same thing was simultaneously designed on two continents, by two men who knew nothing of each other, for different purposes, however. Dr. Rollins intended it for cutting the gums and the periosteum in the transplanting of teeth, while I used it for the cutting out of moles from the skin of the face. I can only admit Dr. Rollins' priority in the invention of the tubular knife for the dental engine, when he can prove that he made a publication of his design prior to April 9, 1884.

PROF. DR. BUSCH,
BERLIN, April 24. Director of the Dent. Inst. of Berlin.

---

*Editor Independent Practitioner:—*

Will you kindly publish in the next issue of your Journal the correct interpretation of the law just passed, amending the Dental Act in this State. Does it throw out of employment all persons now engaged as assistants in operative dentistry? Many of these have been so employed for many years—some since before the passage of the original act of 1879—and to-day they are as competent to perform skillful operations as many of the licensed practitioners. Is it intended to deprive the old assistants of the means of earning a livelihood, and to oblige them to give place to the young and inexperienced student?

Your interpretation of the law will be greatly appreciated by all readers of the PRACTITIONER.

P. M. HARDER,

Ass't in Operative Dentistry.

50 West 35th Street, New York,

May 14, 1888.

ANSWER.

The editor of this Journal does not pretend to be an expounder of the law. He was not consulted in the drafting of the amendment, and has little knowledge of its history, save that it was intended to prevent the practice of unqualified men under cover of the name of a registered dentist. In many cases, those who possessed the necessary certificate have outraged decency and enabled others to violate the plain intent of the law, by a pretence of ownership of a branch office in which a student or other unqualified man was permitted to practice. When suit was brought, the more unscrupulous quack of the two would make oath that the office and practice were his, and the lesser charlatan was his hired assistant. In some instances, a dentist has established a chain of offices in which he has installed his students, and thus practiced quackery by wholesale.

These were the men and this the practice at which the new amendment was aimed. In all the legislation that has been secured, it was the earnest desire of those who advocated it that no vested right should be interfered with, no injustice done to any one. But it is not always possible to get through such a bill as is desired. So many ward caucus statesmen who are in the Legislature must be consulted, that their opposition may not be encountered, each of them knowing infinitely better what is wanted than the parties interested, that by the time they are through with tinkering and patching it is but a selvedge and remnant at best, and the parties are obliged to accept or see it fail altogether.

Nine years should certainly be sufficient time for an assistant who has any interest in his vocation to qualify himself under the law. If he has not done so the fault lies with himself. We think all may rest secure in the assurance that the bill will not be so interpreted as to make it oppressive to any worthy man.

---

### THE ETHICS OF THE MEDICAL PROFESSION.

"Usually the clergy do not comprehend the ethics of the medical profession better than the laity. Generally, the publication of religious as well as secular journals approve of the quack and condemn the honest, regular practitioner. Of the golden rule, as applied to medical men in their professional work, the clergy are, as a rule, utterly ignorant. But a writer in *The Wesleyan Christian Advocate*, while discussing "Ministerial Quackery," makes the following remarks about the medical profession. It will be the more appreciated because of its rarity. It is the first ray of that rising sun which shall one day illuminate the entire world respecting the ethics of medical men :—"

"It is a noteworthy fact that the medical profession has, more perfectly than any other, maintained its high and pure standard of excellency, and all the time made steady and substantial progress. There is something significant in this when we remember that this profession has kept its face against all forms of quackery. It recognizes as a great truth that the interests of humanity demand that a physician and his work should be estimated solely by their real worth. The man who advertises his excellencies, or has himself thus advertised, is suspected at once as being shallow, if not fraudulent, and being conscious of his deficiencies and his inability to pass on his real merit, he resorts to the newspaper as a means to catch patronage from light heads and the unthinking. The code of ethics of this profession is steadily against all humbuggery, and we note with pleasure that meekness and modesty has not yet been retired from the code. We readily see that a man in this profession is thus kept upon that high plane where his eye is ever fixed upon an ideal that is pure and elevated, and worthy of a man."—*American Lancet.*

---

### DR. J. N. FARRAR'S BOOK.

The first volume of the work of Dr. Farrar on "The Correction of Irregularities of the Teeth" was promised the profession before this time, but it has been thought advisable by the author to hold it until the second volume is ready for issue, that it may more nearly appear as a perfect work. The editor of this journal has lately had an opportunity to examine the proof sheets—for the first volume is in type—and he feels assured that nothing will be lost by the delay. The work will be very exhaustive and complete, and its scope such as to cover all, or nearly all, the abnormalities of the human dentition. Particular care has been exercised in tracing out the origin of the various appliances used in the correction of irregularities, and everything of importance is described and illustrated. Indeed, in the matter of cuts the book will be especially rich, there being about 1,400 in the two volumes of 650 pages each.

### OBITUARY.

Died.—In New York City, May 19, 1888, Chauncy P. Fitch, M. D., in the seventieth year of his age. Dr. Fitch was at one time a prominent and well-known member of his specialty. Some years ago he took an active part in society gatherings, but latterly he took very little interest in such matters, seldom meeting with his professional friends. He was born in Vermont, and lost his father when quite a youth. For a time thereafter he worked in the printing office of his uncle, finally taking up the study of medicine and graduating from the University of Pennsylvania. He located in New York a quarter of a century ago, where he has ever since practiced dentistry. He was President of the American Dental Association in 1866, at its meeting in Boston, was one of the founders of the New York College of Dentistry, and aided materially in procuring its charter. C. E. F.

### DENTAL SOCIETY OF THE STATE OF NEW YORK.

The twentieth annual meeting was held at Albany, Wednesday and Thursday, May 9th and 10th. The following members were elected as officers for the ensuing year:—

*President*—J. Edward Line, Rochester.

*Vice-President*—C. F. Rich, Saratoga Spa.

*Secretary*—Myron D. Jewell, Richfield Spa.

*Treasurer*—H. G. Myrick, Brooklyn.

*Correspondent*—G. L. Curtiss, Syracuse,

*Censors*—Second District, Wm. Jarvie, Brooklyn. Fifth District, S. B. Palmer, Syracuse.

Six candidates passed the examination of the Board of Censors and received the degree of Master of Dental Surgery.

### SUSQUEHANNA DENTAL ASSOCIATION.

The twenty-fourth annual meeting of this Society was held at Scranton, Pa., May 16th and 17th. The annual address was made by the President, Dr. J. D. Wingate, of Carbondale. The following were elected officers for the ensuing year :—

*President*—B. F. Van Buskirk.

*Vice-President*—J. L. Fordham.

*Treasurer*—H. Gerhart.

*Recording Secretary.*—V. S. Jones.

*Corresponding Secretary*—C. F. Meaker.

*Executive Committee*—C. S. Beck, J. D. Wingate, H. C. Sticker.

### AMERICAN MEDICAL ASSOCIATION.

The thirty-ninth annual meeting was held in Cincinnati, Ohio, May 8, 9, 10 and 11. The officers elected for the ensuing year are :—

*President*—W. W. Dawson, Ohio.

*First Vice-President*—W. L. Schenck, Kansas.

*Second Vice-President*—Frank Woodbury, Pennsylvania.

*Third Vice-President*—H. O. Walker, Michigan.

*Fourth Vice-President*—J W. Bailey, Georgia.

*Treasurer*—R. J. Dunglison, Pennsylvania.

*Secretary*—Wm. B. Atkinson, Pennsylvania.

*Librarian*—C. H. A. Kleinschmidt, District Columbia.

*Section of Dental and Oral Surgery*—Chairman, F. H. Rehwinkel, Ohio. Secretary, E. S. Talbot, Illinois.

The next meeting will be held at Newport, R. I., the second Tuesday in June, 1889.

---

### IOWA STATE DENTAL ASSOCIATION.

The twenty-sixth annual meeting was held in the Hall of the Dental Department of the Iowa State University, Iowa City, Tuesday, Wednesday, Thursday and Friday, May 1, 2, 3 and 4. President W. P. Dickinson, of Dubuque, presided, and the meeting was one of interest and profit. The following were elected officers for the ensuing year :—

*President*—J. B. Monfort, Dubuque.

*Vice-President*—L. K. Fullerton, Waterloo.

*Secretary*—G. W. Miller, Winterset.

*Treasurer*—F. M. Shriver, Glenwood.

Des Moines was selected as the next place of meeting.

---

### NEW HAMPSHIRE DENTAL SOCIETY.

The twelfth annual meeting of the New Hampshire Dental Society will be held in Concord, June 17th, 1888, at 11 o'clock.

Efforts are being made to have this meeting the best ever held by the society, and all dentists of the State are earnestly requested to be present.

The Board of Censors will meet at 7 o'clock P. M., June 18th, for the examination of candidates for license to practice in the State.

EDWARD B. DAVIS, Secretary,
88 N. Main St., Concord, N. H.

---

### PENNSYLVANIA STATE DENTAL SOCIETY.

The twentieth annual meeting will be held in Philadelphia, commencing Tuesday, June 5th, and continuing three days. Special rates have been secured at hotels and upon the railroads, and a more than usually interesting meeting is confidently anticipated.

La Nature, in an article on "Umbromania," thus speaks of the shape of the hand with reference to manual dexterity :

Thirty-five years of research have permitted M. Etienne, who has been continuously in contact, in shops, with Swiss watchmakers' apprentices, experienced workman, and artists even, to find a certain criterion by which to judge of aptitudes in different trades and several professions.

A young Frenchman who, after reverses of fortune, was desirous of giving up the study of the law in order to learn watchmaking, presented himself one day before M. Etienne at the shop of a skillful master of apprenticeship, who received the intelligent countenance with eagerness; but while pressing the hand of the future apprentice, a cloud passed over the face of the placid master clockmaker. "What did you feel then in pressing the hand of that young man who has just gone out?" asked M. Etienne "With hands like his, we don't make a watchmaker," was the reply, and the prediction came true. It was as a consequence of this conversation that M. Etienne sought and discovered the following rules, that we think we can reproduce without straying from our subject.

The characteristic of dexterity is shown in the first place by *the curve of the thumb arched outwardly*. This is an indispensable condition for the handling of the hammer. The blacksmith who wields with his arm the heavy striking mass that he lets fall perpendicularly, without deviation, repea edly upon the same point, the file cutter, who strikes so regular blows upon the chisel that no flaw is visible in the cut, so equal everywhere is the imprint of the tool—these and all superior workmen, all artists who shape hot iron with the hammer, who chisel the precious metals, who sculpture marble and stone, owe the exact precision in the force and accuracy of the blows that they give with the hammer to the suppleness of the first joint of the thumb. To this natural gift they owe their fortune, for, in shops, selection is made, to the profit of the most skillful, of those alone to whom the most difficult and most delicate work can be entrusted.

A second characteristic of skillfulness is indicated by the faculty of reversing the metacarpal phalanges of the fingers, so that when the hand is extended it is convex. On the greater or less flexibility of all the joints, either at the bone or extremity of the fingers, depend the dexterity and skillfulness displayed in work executed with the file, the plane, or lathe.

This suppleness cannot be independent of that of the thumb, but it does not replace it, while the curved thumb will more easily dispense with the great flexibility of the other fingers. The two characteristics are in most cases united.—*Scientific American.*

It will be remembered that the ingenious Daniel Doyce, in Dicken's "Little Dorrit," turned a spectacle case in his hand "with a certain free use of the thumb, that is never seen but in a hand accustomed to tools."

---

Dr. Williamson exhibited recently to the Odonto-Chirurgical Society of Scotland an interesting case of fracture of the root of a central incisor which bore evidence of having been united. There was a history of a blow in childhood, from which the right incisor received so much injury that its pulp died, as was shown by its discolored appearance. But both teeth had done good service until the patient reached the age of 45, when the left central became so loose that it was removed by the fingers. A part of the root, however, was left behind, but being loose, it was easily extracted. On examination it was found that the two fragments fitted accurately when placed in apposition, except where there was a little chipping at one edge. The fracture of the dentine was at a higher level than that of the cementum, so that the latter formed a sort of collar for the lower fragment There was some thickening in parts of the cementum, and the whole of the pulp in the coronal fragment was calcified, and also the part close to the line of fracture in the other piece.—*London Lancet.*

---

There seems to be almost no end to the new hypnotics, narcotics and local anæsthetics, offered to the medical profession since the discovery of cocaine. The most of them, it is safe to say, are like "Gleditschine," of unsavory memory—made to sell. The latest aspirant has been mentioned by very high authority. *The British Medical Journal* says of it: "Boldin is the glucocide obtained from boldo leaves, and Dr. Junanville highly praises it in a recent number of *Le Progrès Médical*, as a hypnotic far exceeding opium and chloral. This is saying a good deal for it. We are told also that boldin is not disagreeable to take, has no unpleasant effects, increases the appetite, and has a "strengthening" influence on the patient. Between five and ten grains were given daily to various patients The sleep induced by this substance is of a natural kind, and the breathing is regular and tranquil. Boldo leaves contain about three per cent. of boldin. It may be given in capsules in doses of 0.2 grams (three grains), repeated as necessary, or (diluted 1 in 20 in water) subcutaneously."

---

It will give pain to many hearts to know that death in a peculiarly repulsive guise visited the home of the honored Prof. J. Taft, of Cincinnati, on the fourteenth of April last. On that date Mrs. Taft was returning from a visit to her son, and alighted from a street car, when a train upon the Ohio and Mississippi Railroad, which passes the house, came thundering along, struck and hurled her to instantaneous death. Mrs. Taft was devoted to home and its duties—a home which, deprived of its central figure, can never again be what it was. The sympathy of a whole profession is extended to the survivor of this so long united pair, and the wish is fervent that he may not be again called upon to pass through deep affliction.

---

A Law Dispensary has been established in New York, under the auspices of the People's Mission, for the benefit of the poor who require legal advice and cannot afford to pay for it.

What business has a poor man with law? If they would dispense justice, it might be a work of genuine charity.

---

The British Dental Association meets in Dublin this year, and a large attendance is expected. The meeting will be held in August. This is the governing body of the dental profession of Great Britain.

Dr. H. W. Parsons, of Wamego, Kansas, more than a year ago sent the editor of this journal some amalgam for practical tests. It has been possible with it to make fillings that seem as near perfection as can be attained with that material. Some very large contour work bears a polish like that of gold; the color remains excellent, while there is no appearance of shrinkage or drawing away from the walls of the cavity. Other practical tests made with it show quite as good results.

Some large fillings made with an oxy-phosphate cement, prepared by him, have been in the mouth since August last, with apparently no change or disintegration. We have used it for setting gold crowns and for other work, with great satisfaction.

---

We have, in a previous number, spoken of that magnificent work. "Photographic Illustrations of Skin Diseases," published by E. B. Treat, 771 Broadway, New York. We are in receipt of parts five and six, and they fully sustain the high reputation won by previous numbers. The hand-colored plates of Pityriasis, Lichen, Herpes, Zoster, and other skin diseases, are marvelous productions. The work will be completed in twelve parts.

---

"Professor, what are your views concerning the schools of medicine and theology?"

"That depends upon circumstances. When I am slightly ill I am a homœopathist and a Unitarian; but when I am very sick I am an allopathist and a Calvinist."—*Am. Prac. and News.*

---

The attractive side of "Hospital Life" is presented in *Scribner's* for June, by one who looks at it from a patient's point of view. It contains bits of humorous and pathetic character sketching. J. Alden Weir, W. L Taylor, and other skillful artists made the drawings in the New York and Brooklyn hospitals to illustrate it.

---

In the Chemical Laboratory. "Professor, what has become of Appleton? Wasn't he studying with the class last year?" "Ah, yes. Appleton—poor fellow. A fine student, but absent minded in the use of chemicals—very. That discoloration on the ceiling—notice it? Well, that's him."

---

The Southern California Practitioner breaks the record with an account of a quartette medical wedding at Los Angeles, California, as follows:

H. Bert Ellis, M. D.; Lula Talbot, M. D.

F. D. Ballard, M. D.; Rose Talbot, M. D.

---

Drs. G. V. Black and J. W. Wassall, of Chicago, will visit Europe during the summer, and remain abroad for some months. Dr. Black will pursue some special studies during his absence.

---

Philadelphia has four dental journals, according to Caulk's Annual. New York has but one, but that one is the Independent Practitioner.

## Figures and Tables

**Figure f1:**
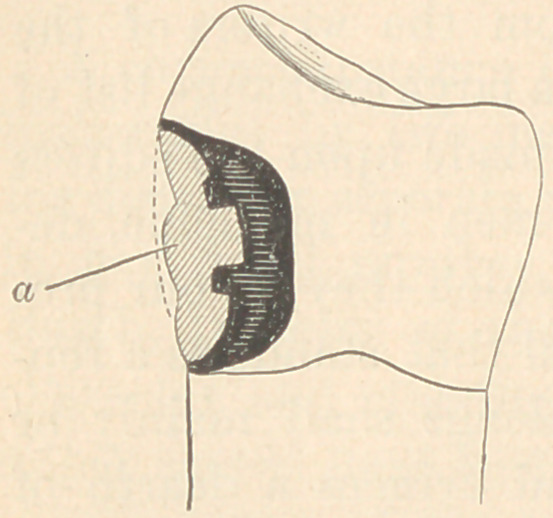


**Figure f2:**